# Calpain Mediated Proteolysis of Junctophilin-1 Produces an Aggregation Prone C-Terminal Fragment in Skeletal Muscle

**DOI:** 10.21203/rs.3.rs-8663093/v1

**Published:** 2026-02-03

**Authors:** Eshwar Reddy Tammineni, Lourdes Figueroa, Carlo Manno

**Affiliations:** Rush University Medical Center; Rush University Medical Center; Rush University Medical Center

**Keywords:** Skeletal muscle, Junctophilin 1 (JPh1), Calpain-1, Protein aggregation, HSP70

## Abstract

Junctophilin-1 (JPh1) is an essential structural protein of the calcium release units required for excitation–contraction coupling in skeletal muscle. In myopathic conditions associated with elevated intracellular calcium, calcium-activated calpains target multiple proteins. Although JPh1 is known to be a calpain substrate, the precise molecular identity of its calpain cleavage sites and the (patho)physiological roles of the resulting proteolytic fragments remain poorly defined. Here, we combined *in-silico* prediction with *in vitro* calpain cleavage analysis of dual-tagged JPh1 to identify multiple calpain cleavage sites within JPh1. We further show that a 44-kDa C-terminal fragment of JPh1 (JPh44) is intrinsically prone to aggregation. Using a combination of biophysical, biochemical, and imaging approaches, we demonstrate that under stress conditions JPh44 progressively forms aggregates that localize predominantly to perinuclear regions. Aggregated JPh44 colocalizes with HSP70 and resides near HDAC6. Pharmacological activation or overexpression of HSP70 promotes clearance of JPh44 aggregates and enhances JPh44 nuclear translocation. Finally, we identify the transmembrane domain of JPh44 as a key determinant driving its aggregation propensity. Together, these findings reveal that stress-induced proteolysis of JPh1 generates aggregation-prone fragments whose handling by heat shock proteins and autophagy-related machinery may play an important role in skeletal muscle adaptation and pathology.

## Introduction

Calcium release from the sarcoplasmic reticulum (SR) into the cytosol during excitation–contraction (EC) coupling in skeletal muscle is fundamental not only for force generation but also for broader muscle biology functions, including metabolic signaling, hormone secretion, and gene regulation^1^. The functional EC coupling machinery is organized by a set of core proteins, including Cav1.1 (DHPR), its auxiliary β subunit, the ryanodine receptor (RyR1), Junctophilin, and STAC proteins, which enable structural membrane communication and calcium flux^2^. Emerging evidence now indicates that under pathophysiological stress, some of these EC coupling components, in either their full-length or proteolytically cleaved forms, can exit the terminal cisternae and translocate to the nucleus, where they function as non-canonical signaling molecules that directly modulate gene transcription^3,4^.

Junctophilins (JPhs) are critical structural proteins that bridge the plasma membrane transverse (T)-tubule and SR membranes by binding to both the T-tubular dihydropyridine receptor (DHPR) and the SR ryanodine receptor (RyR), thereby maintaining the structural integrity of the T–SR junction^5^. Prior studies have demonstrated that both the skeletal muscle JPh isoform (JPh1) and the cardiac JPh isoform (JPh2) undergo proteolytic cleavage in response to elevated intracellular calcium levels and activation of calcium-dependent calpains^6^. Recent findings indicate that calcium activated calpains in malignant hyperthermia–susceptible (MHS) skeletal muscle cleave JPh1^4^, while similar calcium-activated proteases cleave JPh2 in cardiac hypertrophy models^7,8^. Remarkably, proteolytic fragments derived from both JPh1 and JPh2 have been shown to translocate to the nucleus, where they participate in the regulation of gene transcription that help to reduce the stress burden^4,7^.

Genetic mutations and post-translational proteolytic modifications can have profound effects on the structure and function of proteins. Recent studies have demonstrated that structural destabilization of key EC coupling proteins, such as calsequestrin (Casq1) and Junctophilin-2 (JPh2) due to genetic variations and proteolytic processing under myopathic conditions^9,10^. Protein aggregation represents one such destabilizing consequence, often leading to a gain of toxic function. These protein aggregates increase especially when these modified proteins are exposed to additional stress conditions within a diseased cellular environment^11^. To maintain proteostasis, cells deploy multiple quality-control mechanisms to manage misfolded or aggregated proteins. These include molecular chaperones, such as heat shock proteins (e.g., Hsp70), which assist in refolding misfolded proteins, and the autophagy–lysosomal degradation pathway, which facilitates the clearance of insoluble protein aggregates. Dysregulation or overload of these protective systems can exacerbate cellular stress and contribute to the progression of muscle pathology^12^.

In this study, we investigated the proteolytic action of calpain-1 on Junctophilin-1 (JPh1) in vitro and examined the cellular fate of the resulting C-terminal JPh44 fragment. Our results demonstrate that JPh44 exhibits increased aggregation under cellular stress conditions. Aggregated JPh44 colocalizes with the molecular chaperone Hsp70. Pharmacological activation of Hsp70 using BGP-15 or Hsp70 overexpression facilitates aggregate clearance and promotes JPh44 nuclear translocation. Conversely, proteasome inhibition with MG-132 exacerbates JPh44 aggregation and promotes HDAC6 localization in close-proximity towards aggregated JPh44, implicating both chaperone- and autophagy-related pathways in JPh1 fragment turnover. Finally, we identified the transmembrane domain region of JPh44 as a key determinant driving its aggregation propensity. Together, these findings suggest that cellular stress–induced proteolysis of JPh1 generates aggregation-prone fragments whose handling by heat shock proteins and autophagy machinery may play an important role in skeletal muscle adaptation and pathology.

## Results

### Characterization of JPh1 fragmentation products following Calpain-1 proteolysis

**Figure 1:**
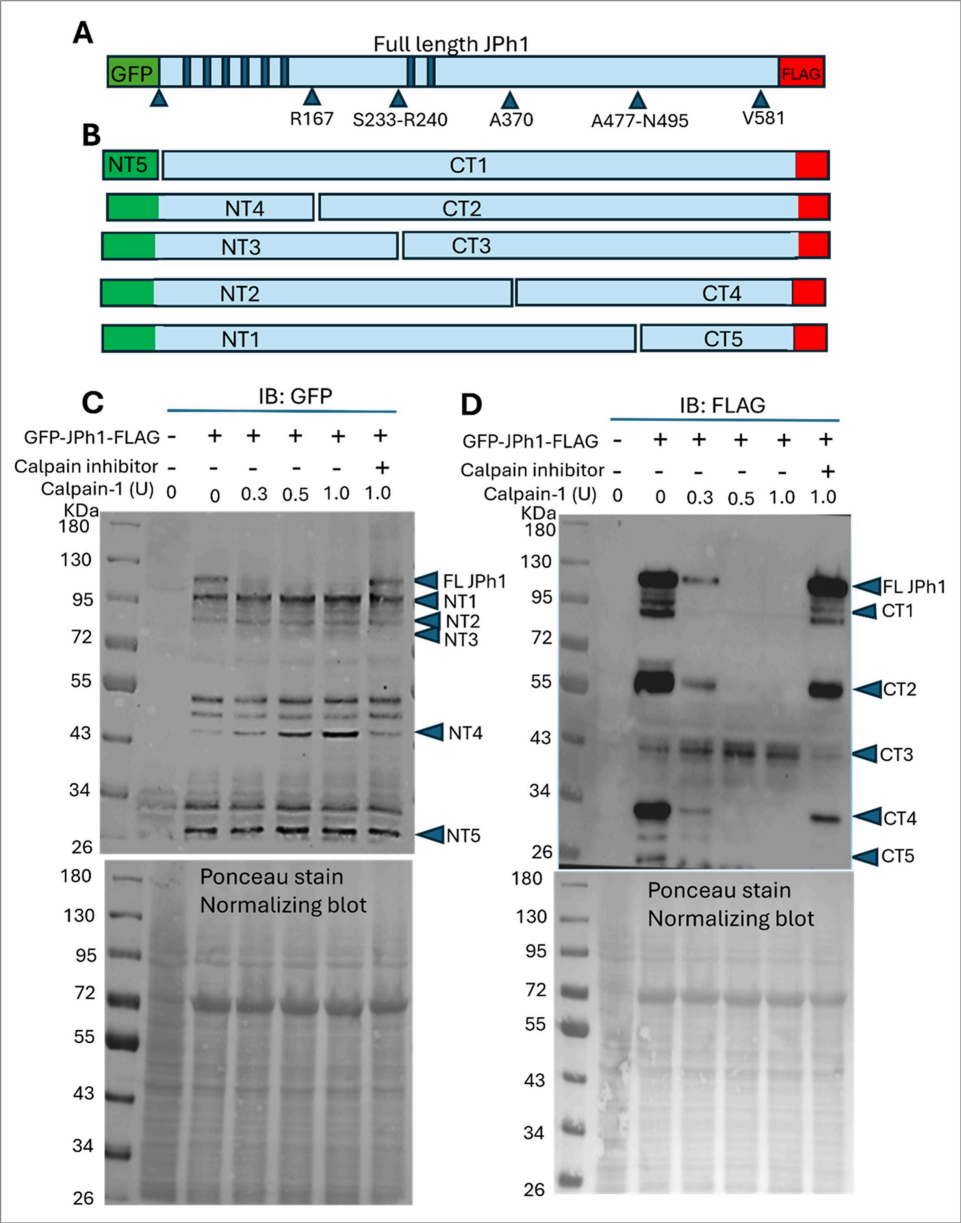
In silico prediction and experimental validation of calpain-mediated JPh1 cleavage. **(A)** In silico prediction of calpain cleavage sites in human JPh1 using the GPS-CCD algorithm identifies multiple putative cleavage sites, particularly between the MORN motifs and within the C-terminal region. **(B)** Schematic representation of JPh1 illustrating the predicted N- and C-terminal cleavage fragments based on the GPS-CCD analysis. **(C)** Representative immunoblot of HEK293 cell lysates expressing GFP–JPh1–FLAG following incubation with increasing concentrations of purified calpain-1, probed with anti-GFP antibody to detect N-terminal cleavage fragments. **(D)** Immunoblot probed with anti-FLAG antibody to detect C-terminal cleavage fragments generated by calpain-1 treatment. Ponceau stained membrane demonstrates uniform protein loading.

Previous studies have reported proteolytic fragmentation of the skeletal muscle junctophilin isoform JPh1^4,6^. However, the full spectrum of JPh1 cleavage fragments and their specific cleavage sites remain incompletely characterized. In silico analysis using the GPS-CCD computational tool predicted multiple potential calpain cleavage sites within JPh1^13^, with prominent clusters located between the MORN motifs and within the C-terminal region (Fig. 1A; Supplementary Table 1). Based on these predictions, plausible N- and C-terminal cleavage products are shown in Fig. 1B. To validate these predictions experimentally, we expressed recombinant human JPh1 tagged with GFP at the N-terminus and FLAG at the C-terminus in HEK293 cells. Cell extracts were exposed to purified calpain-1 in a concentration-dependent manner, and the resulting N- and C-terminal fragments were detected using GFP and FLAG antibodies in western blots (Figs. 1C & 1D). GPS-CCD identified four predominant cleavage regions with the highest predictability scores (> 1.0) (Fig. 1A). In the C-terminal region, four predicted cleavage points—K484, S478, N495, and A477—span a 19-amino-acid divergent segment. Cleavage in this region is expected to generate a long N-terminal fragment (NT1) and a short C-terminal fragment (CT5). Consistent with this, treatment with increasing concentrations of calpain-1 resulted in stable accumulation of the GFP-containing N-terminal NT1 fragment (~ 95 kDa), while low-concentration calpain-1 (0.1 U) produced a FLAG-tagged C-terminal CT5 fragment (~ 25 kDa; Supplemental Fig. 1). At higher calpain concentrations (0.3–1.0 U), the CT5 fragment disappeared, likely due to additional cleavage at V581/H582 or K601 in the extreme C-terminal region, as predicted computationally. Inhibition of calpain activity reversed this effect, resulting in disappearance of NT1 and reappearance of CT5, confirming that the observed proteolysis is calpain-dependent.

The second cleavage site predicted by GPSCCD was located in α-helical region at A370. Cleavage here is expected to generate N-terminal NT2 and C-terminal CT4 fragments. Experimental treatment with increasing calpain-1 concentrations led to stable accumulation of the GFP-containing N-terminal NT2 fragment (~ 85 kDa) and generation of a FLAG-tagged C-terminal CT4 fragment (~ 30 kDa). The increasing calpain concentration might further cleaved CT4 region resulting in its disappearance at high calpain concentration. The third cleavage region was predicted between morn motifs VI and VII consisting of 6 cleavage points (R240/S233/S234/S237/R236/D242) spanning 10 amino acids (Fig. 1A; Supplementary Table 1). Cleavage at this region is expected to generate N terminal NT3 and C terminal CT3 fragments (Fig. 1B). Consistently, experimental calpain-1 treatment resulted in stable accumulation of the GFP-containing NT3 fragment (~ 75 kDa) and a FLAG-tagged C-terminal CT3 fragment (~ 44 kDa) (Fig. 1C–D). Unlike the CT5 and CT4 C-terminal fragments, CT3 gradually increased with rising calpain concentrations and remained stable without undergoing further cleavage at the sites responsible for generating CT4 and CT5. The fourth cleavage site predicted by the computational tool is located between MORN motifs VI and VII at R167 (Fig. 1A). Cleavage at this site is expected to generate N-terminal NT4 and C-terminal CT2 fragments (Fig. 1B). Calpain treatment experiments revealed a stable accumulation of a GFP-containing N-terminal band at ~ 50 kDa, likely corresponding to NT4 (Fig. 1C). Meanwhile, a FLAG-tagged band at ~ 60 kDa was observed, potentially representing the C-terminal CT2 fragment (Fig. 1D). At higher calpain concentrations, CT2 was further cleaved at sites responsible for generating CT1, CT2, or CT3, leading to its disappearance. However, the inhibition of calpain activity restored the CT2 fragment, confirming that its generation is calpain-dependent. Further, the C-terminal 95-kDa fragment (CT1) and the N-terminal 26-kDa fragment (NT5) likely resulted from release of the GFP tag from full-length JPH1 (Fig. 1C–D). Increasing the concentration of calpain did not lead to further proteolysis of GFP, as expected.

### C terminal fragment of JPh1 forms misfolded protein aggregates in human myofibers

Previously, we showed that upon JPh1 cleavage, the C-terminal 44 kDa fragment of JPh1 (JPh44) translocate from its original location at the SR triads to the sarcomere I band and to the nuclei of muscle fibers^4^. Interestingly, characterization of calpain-induced JPh1 proteolysis demonstrated that, among several C-terminal fragments generated, only the JPh44 fragment (CT3 fragment) gradually increases with rising calpain concentrations and remains stable without undergoing further cleavage (Fig. 1C).

**Figure 2:**
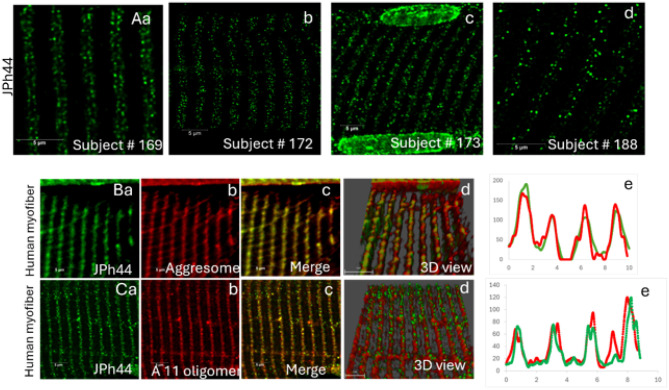
Aggregation and amyloid-like properties of the C-terminal JPh44 fragment in human skeletal muscle fibers. **(Aa–d)** Confocal immunofluorescence images of human skeletal muscle fibers from different subjects stained with a C-terminal JPh1 antibody detecting JPh44 show localization of the fragment to the sarcomeric I band and nuclei. Within the cytoplasm, JPh44 frequently appears in discrete aggregated structures along the I band (arrowheads). **(Ba–c)** Co-staining of human muscle fibers with the JPh44 antibody (green) and Proteostat aggresome dye (red) reveals colocalization of JPh44 aggregates with aggresome-positive structures along the I band, indicating the presence of JPh44 misfolded and aggregated proteins. (Bd–e) Three-dimensional (3D) rendered images of JPh44 (green) and Proteostat aggresome dye (red) and corresponding fluorescence intensity profiles along the I band demonstrate spatial overlap of the two signals. **(Ca–c)** Co-immunostaining of human myofibers with the amyloid oligomer–specific A11 antibody (red) and the JPh44 (green) antibody shows colocalization of aggregated, but not diffuse, JPh44 with A11, consistent with the presence of amyloid-like oligomeric structures in JPh44. **(Cd–e)** 3D renderings of JPh44 (green) and A11 staining (red) along I band and fluorescence intensity profiles across the I band, further confirm colocalization of JPh44 aggregates with amyloid-associated epitope.

To further characterize this fragment, immunofluorescence was performed on human muscle fiber bundles isolated from needle biopsies of different human subjects using a JPh1 antibody raised against a C-terminal epitope (40–51000, Thermo Fisher Scientific), which predominantly detects the C-terminal JPh44 fragment^4^. Figure 2Aa–d shows that JPh44 was observed in the cytoplasmic I band and, in the nucleus, consistent with our previous observations^4^. Upon further examination of the cytosolic distribution of JPh44, we observed that the fragment frequently appeared in aggregated forms along the I band (Fig. 2Aa–d).

To validate the aggregation nature of JPh44, human muscle fibers were co-stained with JPh44 and Proteostat aggresome dye, which binds to misfolded and aggregated proteins. Figure 2Ba–c shows aggregated JPh44 colocalized with Proteostat aggresome dye along the I band, indicating that these JPh44 aggregates are composed of misfolded proteins. Figures 2Bd and 2Be depict the colocalized three-dimensional (3D) rendering of JPh44 (green) and aggresome dye (red), together with their respective fluorescence intensity distribution plots along the I-band. Further, we hypothesized that the misfolded nature of the C-terminal JPh44 fragment arises from an amyloid-prone sequence located within its transmembrane domain (TMD). To test this, human muscle fibers were co-stained with the A11 antibody, which specifically detects generic epitopes associated with the oligomeric state of amyloid proteins, and the JPh1 antibody detecting JPh44. Co-staining experiments in Fig. 2Ca–c found that aggregated, but not diffuse, JPh44 colocalized with the A11 antibody, confirming that the misfolded nature of JPh44 aggregates originates from the amyloid-prone sequence present in the fragment. Figures 2Cd and 2Ce depict the colocalized three-dimensional (3D) rendering of JPh44 (green) and A11 staining (red), together with their respective fluorescence intensity distribution plots along the I-band.

### Palmitic acid induced stress increases the aggregation of C terminal JPh44

JPh44 is enriched in muscles from individuals susceptible to malignant hyperthermia, a condition characterized by elevated basal cytosolic Ca^2+^, increased calpain activity, and heightened oxidative stress^4,14–16^. To investigate the molecular behavior of this disease-associated fragment, we overexpressed a GFP-tagged JPh1 mutant lacking the N-terminal 240 amino acids (GFP-JPH1Δ1–240), corresponding to the C-terminal JPh44 fragment, in C2C12 myoblasts.

**Figure 3:**
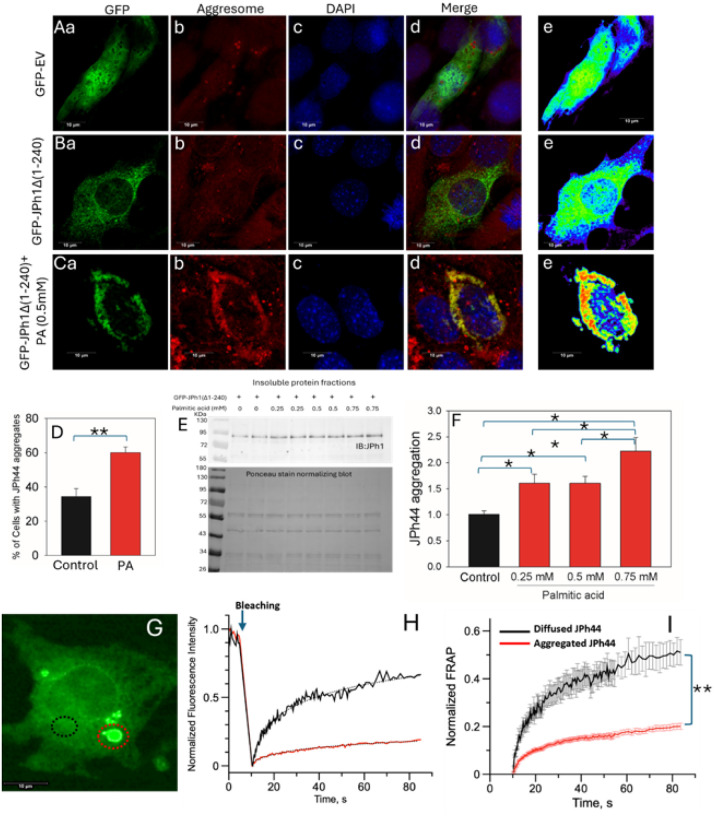
Stress-induced aggregation of GFP-tagged JPh44 in C2C12 myoblasts. **(Aa–e)**Confocal images of C2C12 myoblasts expressing a GFP empty vector showing GFP fluorescence (green), Proteostat aggresome dye (red), nuclei (blue), merged image, and pseudocolored GFP intensity distinguishing diffuse (low intensity; blue/green) from aggregated (high intensity; red) protein. **(Ba–e)**Confocal images of C2C12 myoblasts expressing GFP-JPH1Δ1–240 under basal conditions show cytosolic and nuclear localization of the GFP-tagged protein. Cells are costained with Proteostat aggresome dye (red) and nuclei marker DAPI (blue). Pseudocolored GFP in blue and green highlights predominantly diffuse JPh44. **(Ca–e)** Palmitic acid (PA) treatment increases perinuclear cytosolic aggregation of GFP-JPH1Δ1–240 and its colocalization with Proteostat dye, accompanied by a reduction in diffuse GFP signal in both the cytosol and nucleus. Pseudocolored GFP in red highlights predominantly aggregated JPh44. **(D)** Quantification of cells exhibiting partial or complete GFP-JPH1Δ1–240 aggregation under control and PA-treated conditions shows a significant increase following 0.5 mM PA exposure (34 ± 4.6% Vs 60.5 ± 4.0%; ≥100 cells per condition per experiment; n = 4–5; **p < 0.01). **(E)**Representative Western blot and **(F)** Densitometric analysis of insoluble GFP-JPH1Δ1–240, normalized to total protein by Ponceau staining of the whole membrane, shows a PA concentration-dependent increase in insolubility, with mean ± SEM values of 1.0 ± 0.06 (0.0 mM), 1.6 ± 0.16 (0.25 mM), 1.6 ± 0.13 (0.5 mM), and 2.22 ± 0.26 (0.75 mM) (*p < 0.05). **(G)** Representative live-cell image of GFP–JPH1Δ1–240 prior to photobleaching, with aggregated regions circled in red and diffuse regions circled in black. **(H)**Normalized fluorescence intensity values before photobleaching and fluorescence recovery after photobleaching for aggregated and diffuse GFP–JPH1Δ1–240 regions shown in panel G. **(I)** The mean ± SEM slope values derived from the early phase (at 15 sec) of the FRAP recovery curves were significantly lower for aggregated compared with diffuse GFP-JPH1Δ1–240 regions (0.00748 ± 0.00052 vs. 0.0199 ± 0.0028, respectively; N ≥ 10 aggregated or diffuse regions from multiple cells per experiment; n = 3; **p < 0.01), indicating reduced mobility of the aggregated species.

Under basal conditions, GFP-JPH1Δ1–240 was detected in both the cytosol and nucleus, displaying a combination of diffuse and punctate distributions within the cytosol (Figs. 3Ba and S2). To assess whether the punctate structures represented protein aggregates, cells were co-stained with Proteostat aggresome dye, and GFP fluorescence intensity was pseudocolored, with high-intensity (aggregated) GFP shown in red and low-intensity (diffuse) GFP shown in blue or green. As shown in Figure S2, GFP-JPH1Δ1–240 expression under control conditions exhibited three distinct patterns: (1) completely diffuse localization (Figure S2A), (2) predominantly aggregated protein (Figure S2B) and (3) a mixture of diffuse and aggregated protein (Figure S2C). Notably, only the aggregated, but not the diffuse, cytosolic GFP-JPH1Δ1–240 colocalized with Proteostat dye (Figs. 3Ba–d and S2A–C), indicating that GFP-JPH1Δ1–240 forms misfolded protein aggregates. This behavior is consistent with that of endogenous JPh44 observed in human myofibers (Fig. 2).

To determine whether cellular stress promotes GFP-JPH1Δ1–240 aggregation, cells were treated with palmitic acid (PA), a free fatty acid known to induce the integrated stress response through ER Ca^2+^ dysregulation and oxidative stress^17^. PA treatment markedly increased perinuclear aggregation of GFP-JPH1Δ1–240 and its colocalization with Proteostat dye in the cytosol, while reducing its diffuse distribution in both the cytosol and nucleus (Figs. 3Ca–e). Quantitative analysis revealed that under control conditions, approximately 34% of cells exhibited partial or complete GFP-JPH1Δ1–240 aggregation, whereas PA treatment nearly doubled the proportion of cells containing aggregates (p < 0.01; Fig. 3D). These findings suggest that stress conditions strongly promote GFP-JPH1Δ1–240 aggregation.

Consistent with the imaging data, western blot analysis of insoluble protein fractions demonstrated a concentration-dependent increase in insoluble GFP-JPH1Δ1–240 following PA treatment (p < 0.05; Figs. 3E and 3F). In contrast, expression of a GFP-tagged empty vector in myoblasts, either under control conditions (Figs. 3Aa–d) or following PA treatment (Figure S3), did not result in GFP aggregation. To further distinguish diffuse from aggregated GFP-JPH1Δ1–240 at the biophysical level, fluorescence recovery after photobleaching (FRAP) analysis was performed on regions of diffuse and aggregated GFP-JPH1Δ1–240 within the same cell. As shown in Fig. 3G–I, diffuse GFP-JPH1Δ1–240 regions exhibited rapid and robust fluorescence recovery following photobleaching, indicative of high protein mobility. In contrast, aggregated GFP-JPH1Δ1–240 regions displayed markedly reduced and delayed fluorescence recovery (Figs. 3H and 3I), consistent with limited molecular exchange and immobilization within aggregates. Quantitative FRAP analysis revealed a significant reduction in the slope by 2.6 times in early phase (15 sec) of fluorescence recovery in aggregated regions compared with diffuse regions (Fig. 3I). Together, these findings demonstrate that GFP-JPH1Δ1–240 forms stable, low-mobility aggregates under stress conditions, further supporting its misfolded, aggregation-prone nature.

### Heat shock protein 70 prevents aggregation of C terminal JPh44

**Figure 4:**
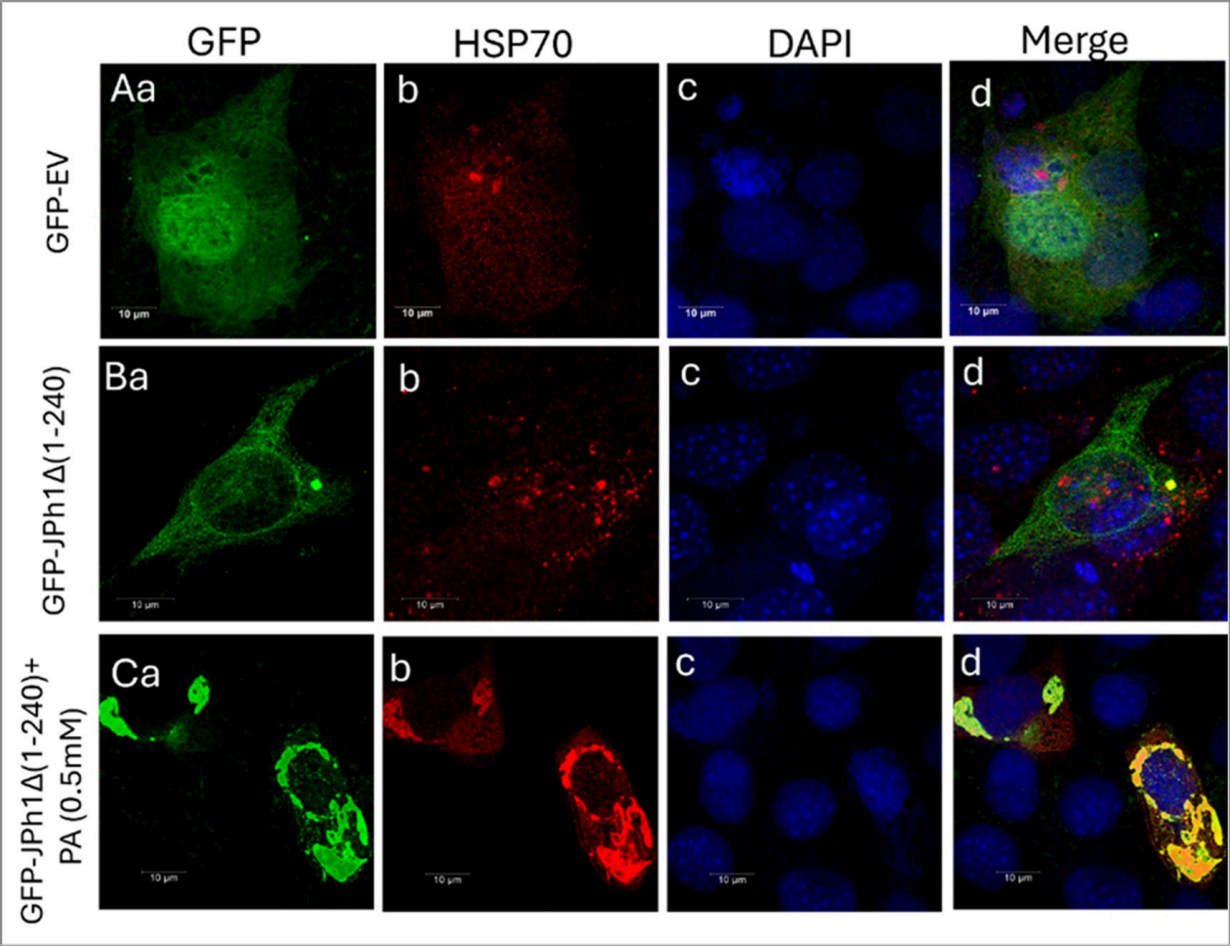
Aggregated JPh44 recruits the cytosolic chaperone HSP70. **(Aa–d)** Confocal images of C2C12 myoblasts expressing a GFP empty vector (green) and immunostained for endogenous HSP70 (red). Nuclei are labeled with DAPI (blue). Merged images show no colocalization between GFP and HSP70. **(Ba–d)** Confocal images of C2C12 myoblasts expressing GFP-JPH1Δ1–240 under basal conditions showing GFP (green), HSP70 (red), nuclei labeled with DAPI (blue), and merged images. Aggregated, but not diffuse, GFP-JPH1Δ1–240 colocalizes with endogenous HSP70 in the cytoplasm. **(Ca–d)** Palmitic acid (PA) treatment increases cytosolic aggregation of GFP-JPH1Δ1–240 (green) and enhances its colocalization with HSP70 (red), predominantly in the perinuclear region surrounding DAPIstained nuclei (blue).

Aggresome formation is known to recruit cytosolic chaperones, such as HSP70, together with proteasomal components, to aggregated proteins, where they recognize misfolded proteins and facilitate their refolding or targeting to proteasomal and autophagic degradation pathways^18^. To determine whether aggregated JPh44 engages this chaperone machinery, we immunostained GFP-JPH1Δ1–240–expressing myoblasts for HSP70. Aggregated, but not diffuse, GFP-JPH1Δ1–240 prominently colocalized with endogenous HSP70 in the cytoplasm (Fig. 4B), whereas cells expressing a GFP-tagged empty vector showed no HSP70 colocalization (Fig. 4A). Palmitic acid (PA) treatment further increased GFP-JPH1Δ1–240 aggregation and enhanced its colocalization with HSP70 (Figs. 4B and 4C), consistent with stress-induced recruitment of heat shock proteins to JPh44 aggregates.

**Figure 5:**
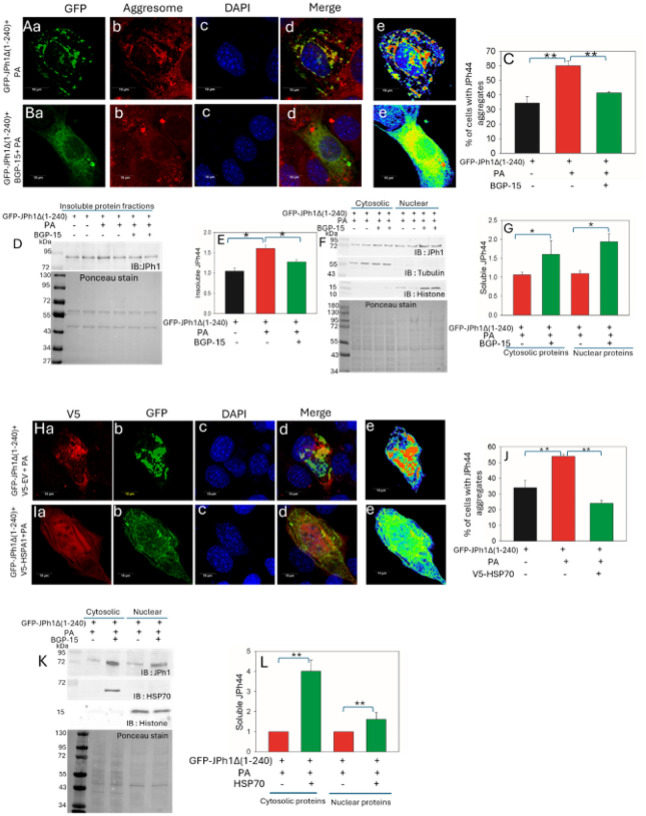
Activation or overexpression of HSP70 reduces JPh44 aggregation and promotes its soluble and nuclear distribution. **(Aa–e)** Confocal images of C2C12 cells expressing GFP-JPH1Δ1–240 treated with palmitic acid (PA) show increased cytosolic aggregation of GFP-JPH1Δ1–240 (green) with perinuclear colocalization with Proteostat aggresome dye (red). Nuclei are stained with DAPI (blue), and pseudocolored GFP highlights aggregated JPh44. **(Ba–e)**Co-treatment with the HSP70 activator BGP-15 increases the diffuse cytosolic pool of GFP-JPH1Δ1–240 and reduces aggregation and Proteostat colocalization; pseudocolored GFP highlights diffuse JPh44. **(C)** Quantification shows that PA significantly increases cells with partial or complete GFP-JPH1Δ1–240 aggregation compared with control, whereas BGP-15 significantly reduces aggregation (mean ± SEM: control, 34.0 ± 4.6%; PA, 60.5 ± 4.0%; PA + BGP-15, 41.0 ± 0.88%; **p < 0.01; n = 3–5). **(D)** Representative immunoblots of insoluble fractions show increased insoluble GFP-JPH1Δ1–240 following PA treatment and reduced accumulation with BGP-15. **(E)** Densitometric analysis normalized to total insoluble protein by Ponceau staining confirms a significant reduction with BGP-15 (mean ± SEM: control, 1.04 ± 0.07; PA, 1.60 ± 0.07; PA + BGP-15, 1.27 ± 0.05; *p < 0.05; n = 4). **(F)** Representative immunoblots of cytosolic and nuclear fractions show GFP-JPH1Δ1–240 levels following PA treatment with or without BGP-15; fraction purity was verified using tubulin and histone markers. **(G)** Densitometric analysis shows that BGP-15 significantly increases soluble GFP-JPH1Δ1–240 levels in cytosolic and nuclear fractions (mean ± SEM: cytosol—control, 1.06 ± 0.06; BGP-15, 1.60 ± 0.35; nucleus—control, 1.10 ± 0.07; BGP-15, 1.93 ± 0.21; *p < 0.05; n = 4). **(Ha–e)**PA-treated cells co-expressing GFP-JPH1Δ1–240 and a V5-tagged empty vector exhibit prominent cytosolic aggregation, whereas **(Ia–e)** co-expression of V5-tagged HSP70 results in a predominantly diffuse cytosolic and nuclear distribution. **(J)** Quantification confirms that HSP70 co-expression significantly reduces aggregation (mean ± SEM: control, 34.0 ± 4.6%; PA, 54.0 ± 1.15%; PA + HSP70, 24.0 ± 1.88%; **p < 0.01; n = 4–5). **(K)**Representative immunoblots show GFP-JPH1Δ1–240 and HSP70 expression in cytosolic and nuclear fractions. **(L)** Densitometric analysis shows that V5-HSP70 overexpression significantly increases soluble GFP-JPH1Δ1–240 levels (cytosol—control, 1.0; V5-HSP70, 4.0 ± 0.56; nucleus—control, 1.0; V5-HSP70, 1.6 ± 0.34; **p < 0.01; n = 3).

To assess whether activation of HSP70 can reduce GFP-JPH1Δ1–240 aggregation, we examined aggregate formation in the presence of the HSP70 activator BGP-15 or following HSP70 overexpression. As observed previously, PA treatment increased cytosolic aggregation of GFP-JPH1Δ1–240 and reduced its diffuse distribution in both the cytosol and nucleus (Fig. 5A). Co-treatment with BGP-15 markedly increased the diffuse cytosolic pool of GFP-JPH1Δ1–240 and promoted its nuclear localization (Fig. 5B). Quantitative analysis revealed that BGP-15 significantly reduced the proportion of cells containing GFP-JPH1Δ1–240 aggregates compared with PA treatment alone, restoring aggregation levels to those observed under control conditions (p < 0.01; Fig. 5C).

Consistent with these imaging results, western blot analysis of insoluble protein fractions showed that PA increased insoluble GFP-JPH1Δ1–240 levels, whereas parallel treatment with BGP-15 significantly reduced the accumulation of insoluble GFP-JPH1Δ1–240. (Figs. 5D and 5E). Ponceau stained membrane of insoluble protein fractions were used to normalize the protein expressions. Subcellular fractionation further demonstrated that BGP-15 co-treatment increased soluble GFP-JPH1Δ1–240 levels in both cytosolic and nuclear fractions compared with PA-treated cells (Figs. 5F and 5G). The relative purity of cytosolic and nuclear protein fractions was verified with anti-tubulin and anti-histone antibodies respectively. Similarly, co-expression of V5-tagged HSP70 completely attenuated the PA-induced increase in GFP-JPH1Δ1–240 aggregation and promoted a diffuse distribution of the protein in both the cytosol and nucleus, compared with cells co-transfected with a V5-tagged empty vector (Figs. 5H–J). Western blot analysis confirmed that HSP70 overexpression significantly increased soluble GFP-JPH1Δ1–240 levels in cytosolic and nuclear fractions under PA treatment (Figs. 5K and 5L).

### Proteasomal inhibition promotes JPh44 aggregation and HDAC6 recruitment

HDAC6 is a key mediator of cellular protein quality control that recognizes ubiquitinated misfolded proteins and facilitates their transport toward perinuclear quality-control compartments, particularly when proteasomal degradation is impaired^19^. To determine whether inhibition of the proteasome enhances aggregation of the JPh44 fragment and promotes engagement of HDAC6-associated pathways, we treated GFP-JPH1Δ1–240–expressing myoblasts with the proteasome inhibitor MG132.

**Figure 6:**
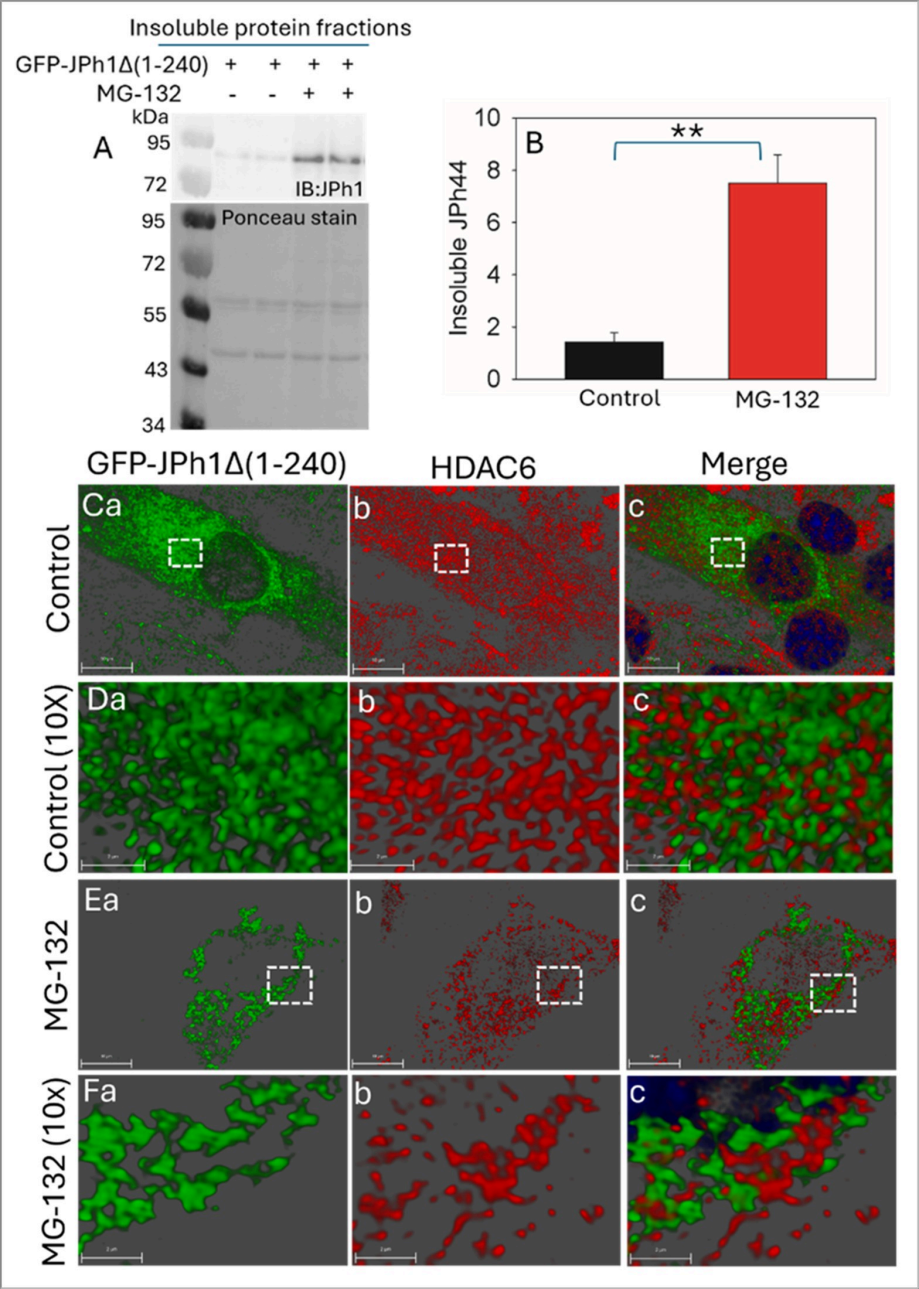
Proteasomal inhibition promotes JPh44 aggregation and its association with HDAC6. **(A)** Representative immunoblot of insoluble JPh44 from C2C12 cells expressing GFP-JPH1Δ 1–240 under control conditions or treated with the proteasome inhibitor MG132. **(B)** Densitometric analysis of insoluble JPh44, normalized to total insoluble protein, demonstrates a significant increase upon MG132 treatment compared with control (mean ± SEM: control, 1.42 ± 0.36; MG132, 7.11 ± 0.94; **p < 0.01; n = 3). **(Ca–c)** Confocal images with three-dimensional (3D) rendering of C2C12 cells expressing GFP-JPH1Δ1–240 (green) and immunostained for endogenous HDAC6 (red) under control conditions show predominantly diffuse GFP-JPH1Δ1–240 with minimal spatial overlap with HDAC6. **(Da–c)** Higher-magnification images of the dashed white boxed region in panel C illustrate the limited colocalization between diffuse GFP-JPH1Δ1–240 and HDAC6 under control conditions **(Ea–c)** MG132 treatment induces prominent perinuclear aggregation of GFP-JPH1Δ1–240 and promotes close spatial association with HDAC6. **(Fa–c)** High-resolution images of dashed white boxed region in panel E highlight the intimate apposition of HDAC6 with MG132-induced GFP-JPH1Δ1–240 aggregates.

Immunoblot analysis of insoluble fractions revealed that MG132 treatment significantly increased insoluble GFP-JPH1Δ1–240 levels compared with control conditions (Figs. 6A and 6B), indicating that proteasomal inhibition promotes the accumulation of aggregation-prone JPh44. To examine the spatial relationship between GFP-JPH1Δ1–240 and HDAC6, we performed immunostaining for endogenous HDAC6 and analyzed protein localization using three-dimensional rendering. Under control conditions, GFP-JPH1Δ1–240 was predominantly diffuse and exhibited minimal spatial overlap with HDAC6 (Figs. 6Ca–c), a pattern also evident at higher magnification (Figs. 6Da–c). In contrast, MG132 treatment induced prominent perinuclear aggregation of GFP-JPH1Δ1–240 assemblies (Fig. 6Ea). Under these conditions, HDAC6 localized in close apposition to the aggregated GFP-JPH1Δ1–240 structures, frequently positioned adjacent to or partially surrounding the aggregates (Figs. 6Ea–c), which is highly evident in high resolution images (Fig. 6Fa–c).

The increased insolubility of GFP-JPH1Δ1–240 and its spatial association with HDAC6 following proteasomal inhibition support a model in which impaired protein degradation promotes JPh44 aggregation and recruitment of aggresome-related machinery.

### Amyloid prone sequence in TMD of JPh44 is responsible for its aggregate formation

**Figure 7:**
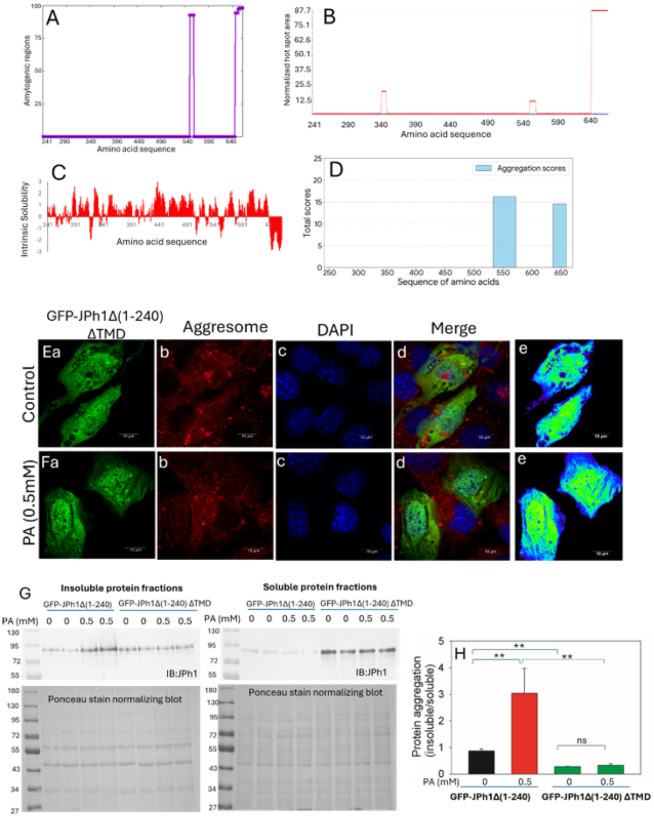
An amyloid-prone transmembrane domain drives JPh44 aggregation. **(A–D)**In silico aggregation-propensity analysis of the JPh44 sequence using four independent prediction algorithms: WALTZ (A), AGGRESCAN (B), CAMSOL (C), and ANuPP (D). All four algorithms identify a common aggregation-prone region overlapping the predicted transmembrane domain of JPh1. **(Ea–e)** Confocal images of C2C12 cells expressing a GFP-tagged JPh44 construct lacking the transmembrane domain (GFP-JPH1Δ1–240ΔTMD) show a diffuse cytosolic and nuclear distribution with no detectable aggregate formation and no colocalization with Proteostat aggresome dye (red). **(Fa–e)** PA treatment does not induce aggregation or Proteostat-positive structures in cells expressing GFP-JPH1Δ1–240ΔTMD. Pseudocolored GFP in green and blue highlights the diffused distribution of JPh44ΔTMD. **(G)** Representative immunoblots of soluble and insoluble fractions from cells expressing GFP-JPH1Δ1–240 or GFP-JPH1Δ1–240ΔTMD under control and PA-treated conditions. **(H)** Quantification of the insoluble-to-soluble ratio shows that deletion of the transmembrane domain (ΔTMD) significantly reduces GFP-JPH1Δ1–240 insolubility under both control and PA-treated conditions (mean ± SEM: GFP-JPH1Δ1–240, 0.87 ± 0.07; GFP-JPH1Δ1–240 + 0.5 mM PA, 3.04 ± 0.92; GFP-JPH1Δ1–240ΔTMD, 0.28 ± 0.01; GFP-JPH1Δ1–240ΔTMD + 0.5 mM PA, 0.33 ± 0.05; **p < 0.01; n = 4**).

To identify the molecular determinants underlying JPh44 aggregation, we performed an in-silico analysis of the JPh44 sequence using four independent aggregation-prediction algorithms—WALTZ, AGGRESCAN, CAMSOL, and ANuPP^20^. Although these tools identified multiple regions with varying aggregation propensities, a single sequence spanning residues G636–T661 was consistently predicted as aggregation-prone by all four algorithms (Figs. 7A–D), indicating a strong intrinsic tendency to promote aggregation. Notably, this region precisely overlaps with the predicted transmembrane domain (TMD; G636–T661) of JPh1 and exhibited the highest aggregation propensity among all predicted segments.

To experimentally validate the contribution of this amyloid-prone TMD to JPh44 aggregation, we generated a GFP-tagged JPh44 construct lacking the transmembrane domain (GFP-JPH1Δ1–240ΔTMD) and examined its subcellular distribution under basal and stress conditions. Confocal imaging revealed that, in contrast to full-length GFP-JPH1Δ1–240, the TMD-deleted mutant displayed a completely diffuse distribution in both the cytosol and nucleus and failed to form puncta or aggregates under control conditions (Figs. 7Ea–e). Importantly, palmitic acid (PA) treatment did not induce aggregation of GFP-JPH1Δ1–240ΔTMD (Figs. 7Fa–e). Consistent with these observations, the TMD-deleted protein showed no colocalization with Proteostat aggresome dye in either the absence or presence of PA (Figs. 7Ea–d and 7Fa–d), indicating that removal of the TMD abolishes aggregate formation.

Biochemical analysis further supported these imaging findings. Western blot analysis of soluble and insoluble fractions demonstrated that the insoluble-to-soluble ratio of GFP-JPH1Δ1–240ΔTMD was significantly lower than that of GFP-JPH1Δ1–240 under both control and PA-treated conditions (Figs. 7G and 7H). Moreover, PA failed to increase the insoluble fraction of the TMD-deleted construct, in contrast to its pronounced effect on full-length GFP-JPH1Δ1–240. Together, these results demonstrate that the amyloid-prone transmembrane domain of JPh44 is a critical determinant of its aggregation and stress-induced insolubility.

## Discussion

MORN motifs located in the N-terminal region of JPh1 anchor the protein to the plasma membrane, while the C-terminal transmembrane domain (TMD) inserts into the sarcoplasmic reticulum (SR) membrane^5^. This configuration positions the non-conserved central region of JPh1 within the cytosol, making it accessible to cytosolic proteolytic enzymes. Our recent studies show that calpain 1, proteases that are activated by cytosolic calcium are enriched at the triads colocalizing JPh1 and RyR1^4^. Their localization near the calcium-release units likely increases their activation probability, as Ca^2+^ released from microdomains during excitation–contraction coupling lowers the threshold for calpain activation. Consequently, calpains positioned at the triad are well placed to cleave the cytosol-exposed central region of JPh1 between the N- and C-terminal anchoring domains. This spatial arrangement of calpains provides a mechanistic explanation for JPh1 cleavage under disease conditions of elevated Ca^2+^ release^4,6,21^. Triad-localized calpains may preferentially target JPh1 during periods of Ca^2+^ leak or sustained Ca^2+^ transients, contributing to structural remodeling of the junctional complex. Such cleavage could alter the stability of the RyR–JPh–DHPR interface and potentially disrupt excitation–contraction coupling efficiency, particularly in pathological states involving Ca^2+^ dysregulation^22^.

Previous studies examining calpain’s effects on JPh2 have reported that its cleavage sites are predominantly located within the C-terminal region^8,23,24^. However, our current findings demonstrate that calpain cleaves both the C- and N-terminal regions of the JPh1. Further, the current study identifies and characterizes the 44-kDa C-terminal fragment of JPh1, which arises due to calpain cleavage of JPh1 between residues S233 and D242. The absence of a similar JPh2 proteolytic product in previous studies and differential effects of calpain on the proteolytic fragmentation patterns of the two junctophilin isoforms can be attributed to the altered sequence homology of JPh1 and JPh2 between the inter-MORN motif regions, as well as the divergent C-terminal domains. Specifically, protein sequence region between MORN motifs 5 and 6 (amino acids 152–280) of JPh1 and region between MORN motifs 6 and 7 (amino acids 152–190) of JPh2 is not conserved. Additionally, the UniProt database predicts that the JPh1 sequence spanning residues 228–247 forms a distorted region, which is inherently more susceptible to proteolytic cleavage than structurally ordered regions^25^.

Correct protein folding is essential for proteins to perform their molecular functions effectively. Failure to maintain a properly folded conformation can lead to pathological abnormalities in muscle diseases^26^. Protein folding and conformation can be disrupted by destabilizing mutations or aberrant post-translational modifications (PTMs), and such misfolding is further exacerbated under pathological stress conditions, leading to the formation of misfolded and aggregated protein species^27^. For example, previous studies have shown that the A405S mutation in junctophilin-2 (JPh2), which is associated with human cardiac disease, leads to the formation of amyloid-like JPh2 aggregates in nuclear and perinuclear regions^10^. Overall, such protein aggregates can impair cellular homeostasis by causing loss of essential protein functions and, in many cases, by exerting toxic gain-of-function effects.

The present study demonstrates that among the proteolytic C-terminal fragments of junctophilin-1 generated by calpain activity, JPh44 is uniquely stable and resistant to further proteolytic processing. In our previous work, we showed that JPh44 is markedly enriched in muscles from myopathic MHS individuals and identified a novel nuclear function for this fragment in regulating gene expression to mitigate cellular stress^4^. Building on these findings, we now show that JPh44 exhibits a strong intrinsic propensity for misfolding and aggregation in the cytosol, particularly under stress conditions that mimic the MHS phenotype. Using Palmitic Acid (PA) treatment to induce oxidative stress, elevated basal Ca^2+^, and insulin resistance^17^—hallmarks of MHS muscle^15,28–30^—we demonstrate that PA induced stress promotes the formation of insoluble JPh44 aggregates that preferentially localize to perinuclear regions. Experiments using a mutant JPh1 engineered to recapitulate the biophysical properties of JPh44 further confirm that this aggregation behavior is intrinsic to the fragment itself. Consistent with a misfolded state, aggregated JPh44 colocalizes with established aggresome markers, including ProteoStat dye and the A11 oligomer-specific antibody, and exhibits markedly reduced mobility by FRAP analysis, indicative of stable, immobile protein assemblies. Importantly, this cytosolic aggregation limits the nuclear translocation of JPh44 and may therefore impair its ability to execute protective transcriptional functions in the nucleus, providing a mechanistic link between cellular stress, JPh44 aggregation, and disrupted stress adaptation in MHS muscle.

Our further results indicate that JPh44 aggregation engages canonical protein quality-control pathways involving molecular chaperones and aggresome-associated machinery. Stress-induced aggregation of JPh44 was accompanied by recruitment of HSP70, consistent with the established role of heat shock proteins in recognizing misfolded proteins and promoting their refolding or delivery to degradation pathways^12^. Supporting this interpretation, previous mass spectrometry–based studies identified an interaction between JPh1 or JPh1 fragment and HSC70, a constitutively expressed chaperone that shares ~ 85% sequence identity with HSP70^31,32^. Pharmacological activation or overexpression of HSP70 markedly reduced JPh44 aggregation, increased its solubility, and restored its diffuse cytosolic and nuclear distribution, indicating that enhanced chaperone capacity can promote the nuclear transcriptional function of JPh fragments and may thereby contribute to stress mitigation. In parallel, inhibition of proteasomal degradation enhanced the accumulation of insoluble JPh44 and promoted its spatial association with HDAC6, a key mediator of aggresome-directed transport under proteotoxic stress^19^. Together, these findings suggest that when proteostasis is overwhelmed, JPh44 is diverted from its nuclear signaling role toward HDAC6-associated perinuclear quality-control compartments, whereas enhanced chaperone activity can partially reverse this process.

We further identified that JPh44, which contains a transmembrane domain (TMD) in its C-terminal region, translocated to the nucleus in our results. Although it remains possible that cleavage could occur within or adjacent to the TMD before nuclear entering, the similar molecular weight of recombinantly expressed nuclear and cytosolic JPh44 suggests that the intact TMD is retained in the nuclear form, indicating TMD-containing JPh44 enters the nucleus rather than a truncated species. Importantly, previous work has shown that JPh44 harbors a nuclear localization signal (NLS) that can drive its nuclear accumulation, consistent with its observed transport into nuclei in muscle fibers and cultured myoblasts^4^. In addition, there is emerging evidence that proteins with TMDs are capable of nuclear translocation through mechanisms that shield hydrophobic segments during nucleocytoplasmic trafficking, with cholesterol playing an important role in facilitating this process^33^. Cholesterol interactions have also been implicated in the function of junctophilin family members, such as JPh2^34^, further supporting a model in which cholesterol-dependent interactions may contribute to the nuclear trafficking of TMD-containing fragments. These findings suggest a multifaceted mechanism for JPh44 nuclear import that involves both intrinsic targeting NLS and lipid-mediated stabilization of hydrophobic domains, aligning with emerging paradigms of unconventional nuclear localization for structurally membrane-associated proteins.

In an attempt to understand the molecular determinants of monomeric JPh44 aggregation, we looked into variety of sequence-based and machine-learning computational tools that have been developed to identify aggregation-prone regions (APRs) within amyloidogenic proteins^20^. Here, we employed four independent algorithms—WALTZ, AGGRESCAN, CAMSOL, and ANuPP—to analyze the JPh44 sequence for intrinsic aggregation propensity. Although each tool identified multiple potential aggregation hotspots, two regions, spanning residues Y550–N558 and G636–T661, were consistently predicted across all four platforms. Notably, the G636–T661 region exhibited the highest amyloidogenic propensity scores in WALTZ, AGGRESCAN, and CAMSOL analyses. Interestingly, this region corresponds precisely to the transmembrane domain (TMD) of full-length JPh1. In support of this, a previous study reported that the TMD facilitates the interaction between two monomeric full length JPh1 molecules, resulting in the formation of a JPh1 dimer^35^. This suggests that TMD present in JPh44 could potentiate interaction among monomeric JPh44’s resulting in an aggregated JPh44. Further, experimental deletion of TMD abolished stress-induced aggregation of JPh44, eliminating proteostat-positive assemblies, and prevented accumulation of insoluble JPh44, demonstrating that the TMD is a dominant structural driver of JPh44 aggregation.

In summary, our findings suggest that calpain-mediated cleavage of JPh1 exposes an intrinsically amyloid-prone transmembrane domain in JPh44, thereby converting this fragment into an aggregation-prone species. Our data further indicates that protein quality-control mechanisms, including molecular chaperones, engage aggregated JPh44 to promote its clearance. Overall, these results reveal a novel molecular mechanism by which elevated intracellular calcium levels and proteolytic activity generate aggregation-prone protein fragments, advancing our understanding of how disrupted calcium homeostasis contributes to pathological protein aggregation and disease phenotypes in skeletal muscle disorders.

## Methods and materials

### Cell culture

Mouse myogenic C2C12 cells and HEK293 cells were obtained from the American Type Culture Collection (ATCC; http://www.atcc.org/) and used up to passage 20. C2C12 cells were cultured in high-glucose Dulbecco’s modified Eagle’s medium (DMEM), whereas HEK293 cells were maintained in standard DMEM, both supplemented with 10% fetal bovine serum (FBS) and 1% penicillin–streptomycin. Cells were grown in a humidified incubator at 37°C with 5% CO2. Culture medium was replaced every 48 h. When cultures reached approximately 70% confluence, cells were either used for transfection or trypsinized for subsequent replating.

### Human muscle biopsies

Human muscle biopsies were obtained from the Malignant Hyperthermia Investigation Unit at the University of Toronto, Canada. Muscle samples used in this study were collected from control subjects or individuals susceptible to malignant hyperthermia (MH). Demographic information for the subjects, organized by identification number and presented in Fig. 2, has been previously reported^4^. All participants provided written informed consent for all aspects of the study, including publication of the data. The use of human tissue and the informed consent language were approved by the Institutional Review Board of Rush University under protocol number 16050502-IRB01.

### Transfections and vectors

Plasmids were transiently transfected into C2C12 myoblasts and HEK293 cells at approximately 70% confluence using either the K2 Transfection System (Biontex Laboratories GmbH, Munich, Germany) or Lipofectamine 3000 (Thermo Fisher Scientific, Waltham, MA, USA), according to the manufacturers’ instructions. Plasmids used in this study included a GFP-tagged empty vector, a dual-tagged JPh1 construct (GFP–JPh1–FLAG), a GFP-tagged construct encoding the JPh44 region (GFP–Δ(1–240) JPh1), and a GFP-tagged JPh44 construct lacking the transmembrane domain (GFP–Δ(1–240) ΔTMD JPh1). All JPh1-related constructs were generated by OriGene Technologies (Rockville, MD, USA). The V5-tagged empty vector (pcDNA5/FRT/TO V5) and V5-tagged HSP70 (pcDNA5/FRT/TO V5 HSPA1A) plasmids were kindly provided by Harm Kampinga (Addgene plasmids #19445 and #19510).

### Invitro JPh1 proteolysis assays

For *in vitro* JPh1 cleavage experiments, HEK293 cells transfected with a dual-tagged JPh1 construct (GFP–JPh1–FLAG) were harvested and homogenized in NP-40 cell lysis buffer (Thermo Fisher Scientific). Cell lysates were centrifuged at 13,000 × g for 10 min, and the resulting supernatants containing soluble protein fractions were used to assess calpain-mediated cleavage of JPh1. Proteolysis was initiated by incubating 50 μg of total protein with 0.3–1.0 μg of purified human erythrocyte calpain-1 (specific activity: 1 U/μg; Millipore Sigma, Burlington, MA, USA) at 30°C for 15 min. Where indicated, reactions were performed in the presence or absence of 10 μg of the calpain inhibitor MDL28170 (Cayman Chemical Co., Ann Arbor, MI, USA), dissolved in DMSO. Reactions were terminated by the addition of SDS sample buffer.

### Immunostaining of human myofibers, and skeletal muscle cell cultures

Immunofluorescence imaging was performed on thin myofiber bundles dissected from human muscle biopsies as described previously^29^, and on cultured C2C12 myoblasts. Human muscle samples were mounted in a moderately stretched configuration in relaxing solution on Sylgard-coated dishes, after which the relaxing solution was replaced with fixative containing 4% paraformaldehyde (PFA) for 20 min. C2C12 myoblasts grown on coverslips were washed with 1× PBS and fixed with 2% PFA for 20 min. Fixed tissues and cell-covered coverslips were transferred to 24-well plates, washed three times for 10 min each in PBS, permeabilized with 0.1% Triton X-100 (Sigma-Aldrich) for 30 min at room temperature, and blocked in 5% goat serum (Sigma-Aldrich) with gentle agitation for 1 h. Primary antibodies were applied overnight at 4°C with gentle agitation, followed by three 10-min washes in PBS. Fluorescent secondary antibodies were then applied for 2 h at room temperature. Samples were mounted using an antifade mounting medium containing DAPI for nuclear staining (ProLong Diamond, Thermo Fisher Scientific). Mounted slides were allowed to cure for at least 24 h at room temperature prior to imaging. Immunofluorescence imaging utilized the following antibodies and aggregate-specific dyes.

### High-resolution immunofluorescence

Immunostained myofibers and cultured cells, as well as cells expressing fluorescently tagged proteins, were imaged using a Leica Falcon SP8 laser scanning confocal microscope (Leica Microsystems) equipped with a 63× water-immersion objective (numerical aperture 1.2). Images were acquired using high-sensitivity hybrid GaAsP detectors (HyD, Leica), allowing low-intensity illumination with minimal photobleaching. Confocal settings included an optimal pinhole size (< 1 Airy unit), extended spectral detection ranges, and acquisition of z-stacks at oversampled x–y–z intervals. Typically, z-stacks consisted of ~ 40 optical sections with 120 nm z-spacing and 60 nm x–y pixel size; for higher-resolution imaging, ~ 20 sections were collected with 120 nm z-spacing and 36 nm x–y pixel size. Dual-channel images were acquired by line interleaving. Most samples were triple-labeled and imaged using excitation/emission settings of 405/430–470 nm, 488/500–550 nm, and 555/570–620 nm. Image acquisition was initiated at the plane closest to the objective, corresponding to or just above the lower surface of the myofiber.

### Fluorescence Recovery After Photobleaching measurements

Fluorescence recovery after photobleaching (FRAP) experiments were performed as described previously^36^ to assess the mobility of GFP–Δ(1–240) JPh1 in C2C12 myoblasts. Cells were plated on 35-mm glass-bottom dishes and transfected with GFP–Δ(1–240) JPh1. FRAP experiments were conducted at the indicated time points using a Leica TCS SP8 confocal microscope at room temperature. GFP fluorescence was imaged using a 488-nm argon laser with a 63× water-immersion objective. Regions of interest (ROIs) corresponding to either diffuse or aggregated GFP–Δ(1–240) JPh1 were selected within the cytosol. Baseline fluorescence was recorded for 5–10 frames prior to photobleaching. Photobleaching was performed by applying a high-intensity 488-nm laser pulse to the selected ROI. Fluorescence recovery was subsequently monitored at low laser power at defined intervals for 90 seconds. Fluorescence intensities were quantified using DataGraph software, background-subtracted, and normalized to pre-bleach values (scaled between 0 and 1). Recovery curves were generated by plotting normalized fluorescence intensity as a function of time. At least 10 ROIs from multiple cells were analyzed per condition in each experiment, with a minimum of three independent experiments performed. Data are presented as mean ± SEM.

### Total cell proteins, Subcellular fractionization and Western blot analysis

Following cell transfection and experimental treatments, cells cultured in Petri dishes were washed three times with 1× PBS. For preparation of total cell lysates, cells were incubated on ice with an appropriate volume of NP-40 Cell Lysis Buffer supplemented with protease and phosphatase inhibitor cocktails (Thermo Fisher Scientific, MA, USA) for 5 min, after which cell extracts were collected using cell lifters. Lysis was continued for an additional 30–40 min on ice, with vertexing every 10 min. Cell lysates were centrifuged at 13,000 × g for 10 min at 4°C, and supernatants containing soluble protein fractions were collected for downstream analyses. Insoluble protein fractions were obtained by resuspending the remaining pellets in 8 M urea solution (Millipore Sigma, MA, USA). Cytosolic and nuclear protein fractions were prepared using the NE-PER Nuclear and Cytoplasmic Extraction Kit (Thermo Fisher Scientific, MA, USA) according to the manufacturer’s instructions. Protein concentrations were determined using the BCA protein assay kit (Thermo Fisher Scientific, MA, USA).

For immunoblotting, protein samples were mixed with Laemmli sample loading buffer (Bio-Rad, CA, USA) and boiled for 5 min. Proteins were separated by SDS–polyacrylamide gel electrophoresis using 4–20% gradient Mini-PROTEAN TGX gels (Bio-Rad, CA, USA) and transferred onto nitrocellulose membranes (Bio-Rad, CA, USA) using a semi-dry transfer system. Membranes were blocked with 4.5% blotting-grade blocker (Bio-Rad, CA, USA) in PBS and incubated with primary antibodies overnight at 4°C. Membranes were subsequently washed with PBS containing 0.1% Tween-20 (Millipore Sigma, MA, USA) and incubated with horseradish peroxidase–conjugated anti-mouse or anti-rabbit secondary antibodies (Invitrogen, Thermo Fisher Scientific, CA, USA) for 1 h at room temperature. Immunoreactive bands were visualized using an enhanced chemiluminescent substrate (Millipore Sigma, MA, USA) and detected with the Syngene PXi imaging system (Syngene USA Inc. MD, USA). Quantitative analysis of Western blot signals was carried out using the Syngene image analysis software (Syngene USA Inc., MD, USA). Total protein levels in each lane, visualized by Ponceau S staining of membranes, were used for normalization. Immunoblotting and immunofluorescence analyses were performed using the following primary antibodies and fluorescent reagents: junctophilin-1 (PA5–52639 and 40–51000, Thermo Fisher Scientific; H00056704-M04, Abnova), β-tubulin (T52011, Sigma-Aldrich), histone H3 (4499, Cell Signaling Technology), HSP70 (SMC-100, StressMarq), GFP (TA180076, Origene), FLAG (NBP1–06712, Novus Biologicals), HDAC6 (NBP1–69127, Novus Biologicals), and anti-amyloid oligomer A11 antibody (SPC-506D, StressMarq). Protein aggregates were detected using the ProteoStat^®^ Aggresome Detection Kit (ENZ-51035, Enzo Life Sciences).

### Statistical analysis

All statistical analysis were performed using SigmaPlot for Windows or DataGraph for Mac. Data are presented as mean ± standard error of the mean (SEM). Differences between two groups were assessed using two tailed Student’s t-test, with a significance threshold of p < 0.05. When data did not meet normality (Shapiro–Wilk test) or equal variance assumptions, the nonparametric Mann–Whitney Rank Sum test was applied. For comparisons involving more than two groups, one-way ANOVA was used followed by appropriate post hoc tests (e.g., Tukey’s or Holm–Sidak) when data met normality and equal variance assumptions; if these assumptions were violated, a Kruskal–Wallis test with post hoc pairwise comparisons was applied.

### Study Approval

This study was approved by the Research Ethics Board of Toronto General Hospital (TGH). Written informed consent was obtained from all patients whose muscle biopsies were used in this study. The consent, which was also approved by the Institutional Review Board of Rush University, permitted the use of biopsy samples for imaging studies. Additionally, all experiments in this study were performed in accordance with relevant guidelines and regulations.

## Supplementary Material

Supplementary Files

This is a list of supplementary files associated with this preprint. Click to download.

- TamminenietalSupplementaltableandfigures2026.docx
- UncroppedblotsTamminenietal.2026.docx

## Data Availability

All data generated or analyzed during this study are included in this article. The data used and/or analyzed during the current study are available from corresponding authors upon reasonable request.
